# Does achieving apical patency increase postoperative pain during endodontics?

**DOI:** 10.1038/s41432-024-01058-8

**Published:** 2024-10-10

**Authors:** Rachel Strickland, Luca Licheri, David Edwards

**Affiliations:** 1 Newcastle Dental Hospital, Claremont Road, Newcastle upon Tyne, NE2 4AZ UK; 2 King’s College Hospital NHS Foundation Trust, Denmark Hill, London, SE5 9RS UK; 3 School of Dental Sciences, Newcastle University, Framlington Place, Newcastle upon Tyne, NE2 4BW UK

**Keywords:** Root canal treatment, Endodontic files

## Abstract

**Data sources:**

PubMed, Scopus, Embase, Web of Science, Cochrane Library, and gray literature up to May 2023. Twelve studies were included for meta-analysis.

**Study selection:**

Two authors screened databases to identify suitable studies, with a third investigator resolving disagreements. Inclusion criteria were randomized controlled trials (RCTs) involving individuals without systemic diseases, who underwent root canal treatment in a permanent tooth with or without apical patency. Outcomes included pain, number of patients with pain and analgesic use. Exclusion criteria included patients who had undergone tooth extraction, incomplete data reports and studies which were not RCTs. There were no limits on publication language. A search of PubMed, Scopus, Embase, Web of Science, Cochrane Library, and gray literature from Google Scholar, ProQuest, OpenAire, and BASE identified 92 articles until May 2023. After removing 42 duplicates, 50 article full texts were assessed. Ultimately, 12 studies were included, seven of which were new relative to previous systematic reviews.

**Data extraction and synthesis:**

Data were analysed using RevMan 5.4 software. Dichotomous categorical data were analysed through the Cochran-Mantel-Haenszel test, with the inverse variance method used for continuous data. Following assessment of heterogeneity with the *χ*^2^ test, fixed- or random- effects modeling was used. Pain scores were reported within one week by ten studies, but three were excluded from meta-analysis due to methodological issues.

**Results:**

Studies involving participants aged 14–65 years, with pulp necrosis, apical periodontitis, or pulpitis, were included for analysis. Irrigation solutions varied from saline to sodium hypochlorite (2.25–5.25%), EDTA, citric acid, or chlorhexidine. Types of teeth treated included molars and anterior teeth. All studies used a 10# K-file for apical patency and various hand and nickel-titanium instruments for canal preparation. Generally, there was slightly reduced post-operative pain reported through the maintenance of patency. This was evident in the initial post-operative period of 24 h (OR = 1.69, *P* = 0.002), 1-day (MD = −1.69, *P* = 0.03) and 2-days (MD = −0.85, *P* = 0.04), but became non-significant over the remainder of the 7-day monitoring period. There was no significant difference in analgesic use between groups (OR = 0.82, *P* = 0.42). Studies were classified into three groups based on quality: four high quality with low risk of bias; three moderate quality with some concerns; and five low quality with high risk of bias. Evidence quality varied; pain scores on days 1 and 2 were of low quality, with very low quality for 12 h and days 3–6. High heterogeneity and imprecision were noted, especially in pain measurements and analgesic use.

**Conclusions:**

The meta-analysis indicates that during the initial post-operative period, apical patency significantly reduces postoperative pain. However, the findings should be interpreted cautiously due to high heterogeneity and variable evidence quality. Given that apical patency is seen as desirable for achieving the biological aims of endodontic treatment, the fact that it also appears to reduce post-operative pain helps to align patient reported outcome measures with traditional measures indicating success.

## A Commentary on

**Xiqian L, Ying Z, Mian M**.

The effect of apical patency on postoperative pain following endodontic therapy: A systematic review and meta-analysis. *Eur J Oral Sci* 2024; 10.1111/eos.12986.

**GRADE Rating:** 

## Commentary

This systematic review and meta-analysis shows that apical patency significantly mitigates postoperative pain during the initial days following endodontic therapy. By using a #10 K-file, dentists can prevent dentin plug formation, ensure better disinfection, and reduce debris extrusion and infection risk. Ultimately, the main aim of endodontic treatment, thorough chemo-mechanical disinfection, is more likely through the maintenance of patency, resulting in superior outcomes^1–4^.

Overall, this was a comprehensive and robust systematic review. A thorough search strategy through databases like PubMed, Scopus, Embase, Web of Science, and the Cochrane Library, and including gray literature was undertaken. No forward or reverse reference searching was undertaken, potentially excluding relevant studies that might affect the outcomes. Furthermore, the selection process, although robust, does not clearly indicate which stages involved two reviewers—whether during title and abstract screening or full-text screening—leaving some ambiguity about the review process.

Other (unavoidable) limitations in the present study include the high heterogeneity of pain scores and variations in study quality, necessitating cautious interpretation of the results. More high-quality RCTs are needed to confirm these findings.

Several potentially confounding variables were not considered in the analysis. This includes the type and concentration of irrigant, hand or rotary/reciprocating instrumentation, size of apical stop, presence of pre-operative pain, presence of pre-operative periapical lesion, number of visits, interappointment medicament, obturation method. Such variables may be key determinants of both success and post-operative pain and discomfort^4,5^.

All included studies have an element of bias, as it would not be ethical or practical to aim deliberately for non-patent root canal treatments, thereby favoring one treatment approach over the other. This introduces a selection bias, particularly since cases with “no patency” would inherently face significant issues in terms of disinfection. It may therefore be that cases with no patency were ‘self-selecting’ due to the complexity of their apical anatomy^6,7^. Additionally, the inclusion of mostly low to medium-quality studies underscores the need for more rigorous research designs.

The overall pain scores in the included RCTs were quite low, with a pain score of 2.2 at 12 h, which reduced daily up to seven days, reaching as low as 0.01. These findings, while promising, need to placed into context with the minimal clinically important difference (MCID) to gauge whether the observed reductions in pain are significant enough to impact clinical practice^8^. A statistically significant difference in pain score may not translate to a clinically important difference.

Despite these limitations, the practice points from this meta-analysis provide actionable insights for improving patient outcomes through effective pain management strategies. Understanding the diverse factors contributing to postoperative pain allows for a personalized approach to endodontic treatment. The preference for rotary instruments and potential benefits of single-visit treatments are practical considerations for optimizing patient care.

In conclusion, while evidence supports the efficacy of apical patency in reducing postoperative pain, ongoing research and well-designed studies are essential to address existing gaps and enhance reliability. Integrating these practice points can improve clinical outcomes and patient satisfaction.

Practice points

- **Implement apical patency**: It does not increase postoperative pain and may help reduce pain within the first 24–48 h.
- **Use #10 K-file beyond ‘0-reading’ in between larger files**: Effective in maintaining apical patency and reducing debris.
- **Tailor pain management**: Consider patient demographics and tooth type.
- **Assess pain accurately**: Use standardized tools and consider analgesic use.

## References

[1] Nair PN. On the causes of persistent apical periodontitis: a review. Int Endod J. 2006;39:249–81. 16584489 10.1111/j.1365-2591.2006.01099.x

[2] Siqueira JF Jr. Microbial causes of endodontic flare-ups. Int Endod J. 2003;36:453–63. 12823700 10.1046/j.1365-2591.2003.00671.x

[3] Goldberg F, Massone EJ, Saita M. Clinical influence of apical patency on the success of endodontic treatment. Endodontic Practice Today. 2010;4:261–6.

[4] Gulabivala K, Ng YL. Factors that affect the outcomes of root canal treatment and retreatment-a reframing of the principles. Int Endod J. 2023;56:82–115. 36710532 10.1111/iej.13897

[5] Ng YL, Mann V, Gulabivala K. A prospective study of the factors affecting outcomes of nonsurgical root canal treatment: part 1: periapical health. Int Endod J. 2011;44:583–609. 21366626 10.1111/j.1365-2591.2011.01872.x

[6] Alves VO, Ahmed HMA, De-Deus G, Silveira CFR, Coutinho-Filho T. Effect of apical patency on postoperative pain in patients undergoing root canal treatment: a systematic review and meta-analysis. J Endod. 2018;44:370–5.

[7] Xiqian L, Ying Z, Mian M. ‘The effect of apical patency on postoperative pain following Endodontic therapy: a systematic review and meta‐analysis’. Eur J Oral Sci. 2024;132:e12986. 38632110 10.1111/eos.12986

[8] Olsen MF, Bjerre E, Hansen MD, Hilden J, Landler NE, Tendal B, et al. Pain relief that matters to patients: systematic review of empirical studies assessing the minimum clinically important difference in acute pain. BMC Med. 2017;15:35. 28215182 10.1186/s12916-016-0775-3 PMC5317055

